# Text mining of verbal autopsy narratives to extract mortality causes and most prevalent diseases using natural language processing

**DOI:** 10.1371/journal.pone.0308452

**Published:** 2024-09-19

**Authors:** Michael Tonderai Mapundu, Chodziwadziwa Whiteson Kabudula, Eustasius Musenge, Victor Olago, Turgay Celik

**Affiliations:** 1 Department of Epidemiology and Biostatistics, School of Public Health, University of the Witwatersrand, Johannesburg, South Africa; 2 MRC/Wits Rural Public Health and Health Transitions Research Unit (Agincourt), Johannesburg, South Africa; 3 National Health Laboratory Service (NHLS), National Cancer Registry, Johannesburg, South Africa; 4 Wits Institute of Data Science, University of The Witwatersrand, Johannesburg, South Africa; 5 School of Electrical and Information Engineering, University of The Witwatersrand, Johannesburg, South Africa; King Caesar University, UGANDA

## Abstract

Verbal autopsy (VA) narratives play a crucial role in understanding and documenting the causes of mortality, especially in regions lacking robust medical infrastructure. In this study, we propose a comprehensive approach to extract mortality causes and identify prevalent diseases from VA narratives utilizing advanced text mining techniques, so as to better understand the underlying health issues leading to mortality. Our methodology integrates n-gram-based language processing, Latent Dirichlet Allocation (LDA), and BERTopic, offering a multi-faceted analysis to enhance the accuracy and depth of information extraction. This is a retrospective study that uses secondary data analysis. We used data from the Agincourt Health and Demographic Surveillance Site (HDSS), which had 16338 observations collected between 1993 and 2015. Our text mining steps entailed data acquisition, pre-processing, feature extraction, topic segmentation, and discovered knowledge. The results suggest that the HDSS population may have died from mortality causes such as *vomiting, chest/stomach pain, fever, coughing, loss of weight, low energy, headache*. Additionally, we discovered that the most prevalent diseases entailed human immunodeficiency virus (HIV), tuberculosis (TB), diarrhoea, cancer, neurological disorders, malaria, diabetes, high blood pressure, chronic ailments (kidney, heart, lung, liver), maternal and accident related deaths. This study is relevant in that it avails valuable insights regarding mortality causes and most prevalent diseases using novel text mining approaches. These results can be integrated in the diagnosis pipeline for ease of human annotation and interpretation. As such, this will help with effective informed intervention programmes that can improve primary health care systems and chronic based delivery, thus increasing life expectancy.

## Introduction

The United Nations expects all countries in the world to meet their 90 percent death registration coverage requirement. However, this is not the case as most deaths that occur in Low to Medium Income Countries (LMICs) are not captured in civil registration systems [1, 2]. As such, this accounts for approximately 65 percent of the world population that do not have a medically certified cause of death (CoD) on a yearly basis [3]. Nevertheless, the CoD information is important as it is used to improve civil registration systems, public health monitoring, critical health policies and priorities [4]. In most instances were CoD cannot be derived from clinically based sources, the VA process is used as a substitute tool. The VA process is conducted by non-medical staff who seek to elicit information from the next of kin of the deceased, regarding circumstances and events that may have led to death [3]. This information is then captured and stored in textual format and is also known as VA narratives. These narratives contain rich valuable information that is used to supplement responses from the standard World Health Organisation CoD questionnaire by physicians, when giving a CoD diagnosis [3]. However, in most cases this information is encapsulated in the textual format, usually unstructured and is stored in large volumes. Consequently, it is difficult to manage, takes time to process and suffers from word polysemy, amongst many challenges. This therefore makes the analysis and harnessing of meaningful information difficult, even though it is vital in identifying mortality causes.

As such, the application of text mining (TM) which employs natural language processing (NLP) in an automated fashion can be beneficial to extract meaningful information regarding mortality causes and most prevalent diseases from VA narratives. There is scanty literature on the application of unsupervised TM approaches within the VA domain. Only a few TM studies that employ supervised learning, have been applied to date within the VA domain to determine CoD [5–7]. Mujtaba et al. [5] argues that the TM approaches used for cause of death classification, require large datasets which are labelled for training the machine learning models. This makes the process tedious, time consuming, expensive and prone to human error as the experts are the ones responsible for labelling the datasets [5, 8].

This study seeks to address this gap by reducing the need for human intervention, by implementing unsupervised TM approaches. The implementation of TM approaches in VA to determine mortality causes and identify the most prevalent disease has several advantages as reported in [5, 6]. Moreover, several studies within the healthcare domain also appraise on the benefits of applying TM as cited in [9–16].

Firstly, TM can help in efficient analysis, through extracting vital information from large volumes of unstructured narratives in a quick, cheaper and accurate way [5]. Ultimately, this reduces time and effort required for manual analysis. Moreover, the TM techniques exhibit the ability to transform unstructured data into actionable insights that can help improve health outcomes, inform public health policies and interventions. The TM mathematical approaches are reported elsewhere [17]. Secondly, implementing TM techniques can result in improved accuracy as one can identify patterns and trends in the data that might be missed by human analysts, leading to more accurate and reliable results [12, 15]. Thirdly, there is consistency as one can categorise similar cases together, ensuring a consistent way of CoD categorisation across different VA records. Additionally, there is some form of standardisation on the CoD categorisation, that ensures the application of the same criteria across different VA records. Interestingly, TM approaches can extract additional variables that can be useful in the CoD diagnosis [12]. Moreover, the semantic and structure analysis through sequential modelling, can even give physicians better insights on the most probable CoD as it entails sequences of events that might have led one to succumb to death [18]. As such, this might help in cases where there are fewer symptoms or unknown CoD to help one to get to a CoD diagnosis.

In this study we present novel TM techniques such as n-gram natural language processing, LDA and BERTopic as we seek to investigate mortality causes and the most prevalent diseases that led to death in rural north-east South Africa. The TM approaches will assist in automating the process of analysing VA data, in a fast and accurate manner. The findings of this study can be integrated in the diagnosis pipeline for ease of human annotation and interpretation. By incorporating modelling frameworks, researchers can potentially improve the granularity and precision of identified topics, leading to a more nuanced understanding of the underlying themes present in large VA text corpora. Ultimately, the discovered knowledge from the unstructured narratives will enable the design, implementation and sustainment of tailored health interventions programmes rather than adhoc generalizations. This will strengthen health priorities, improve life expectancy, VA reporting and decision making, thus informing policy and practice.

## Literature review

### Related work

There is scanty literature that reports on the application of TM techniques specifically in the VA domain. TM is an approach that seeks to close the gap between textual information and representation of the text in a structured fashion [15]. Leskovec et al. [19] argues that the mining of data entails use of very robust hardware, programming languages that employ efficient algorithms, and strive to solve various problems in domain specific areas. TM employs various techniques that span from NLP, data mining, machine learning (ML) and management of knowledge. TM can be used for information retrieval, information extraction, classification of documents and named entity recognition (NER), amongst many applications [14].

One notable study in the VA domain that has applied TM is that of Mujtaba et al. [5], who did a systematic literature review on clinical text classification trends using 72 articles. They point out that the free-text clinical report is very valuable for classification problems. Nonetheless, one can only derive meaningful information from such reports after effective data transformation, to generate useful trends and patterns. Similar work on text classification is reported in [20–23].

Despite the little research that reports on application of TM methods in the VA domain, these approaches have been employed extensively in other health fields as reported in [13, 24–29]. These previous studies report that TM techniques make it possible to extract meaningful information from implicit information. As such, there is great need to use novel TM tools to extract and discover valuable information. Consequently, TM techniques, can be used as alternatives to manual processes. However, they raise issues of noise and redundancy that affect the quality of data. Moreover, in most cases unstructured textual data exhibits high levels of noise, sparsity, makes use of varying vocabulary terms, misspelled terms with many grammatical errors and entails native terms [5, 27, 30].

Recent developments in technology have triggered an increase in the application of electronic public health repositories, making most textual reports readily available for additional use, specifically in biomedicine to extract useful information [5, 29]. However, despite these availed opportunities, there is little to none regarding VA literature that has applied these novel TM techniques for knowledge discovery. On the contrary, VA narratives entail rich valuable information, which can be utilised through NLP, and can be integrated in intelligent models to improve public health decision making processes [3, 15].

### N-gram language processing

Literature reports on n-gram language processing being a feature extraction approach, that aims at attaining only relevant and useful features from textual data. The n-grams are a set of words that are sequential as they make use of continuous number of items such as characters or words from a given sequence of narratives [5, 31]. Another study by Danso et al. [6] reports on a comparative study of machine learning methods for VA text classification and they deduce that the bag of words using n-grams can result in better model performance with high accuracies.

Lucini et al. [14] did a study using TM techniques as they sought to predict hospital admissions using early medical records from the emergency department. They report that these tools make it possible to elicit valuable information, thereby improving clinical decision making processes. Similar findings are also reported in Kim and Chung [16], who conducted a study on information extraction from big health data and they suggest that word clouds can be effective visualisation tools that can be able to depict keywords or concepts in documents. Furthermore, they argue that, the more a term appears in the corpus repeatedly, the more important it is. Similar work which emphasises the importance of n-grams as a TM approach in gaining valuable insights is alluded in the studies of [32–34]. However, they point out the challenges of having too many insignificant words in the corpus.

### Topic modelling

#### Topic modelling using LDA

Topic modelling entails using probabilistic algorithms to extract useful and common topics from a set of documents (in our case we aim to extract most prevalent diseases). Topic modelling is an unsupervised learning approach based on statistical principles, that seeks extract topics from a set of documents stored in given corpus. A single topic is made up of a collection of words, with a corresponding weight. A single word can belong to various topics and documents can be made up of several topics [35].

One commonly used topic segmentation method is the LDA, which has been applied in domains such as classification of genomic sequences, social networks, modelling of health data, extracting latent topics from clinical reports, amongst many uses. This approach has been reported to attain high precision and performance scores, especially when used with clinical reports [10, 35–38]. LDA is generative in nature and can easily pick out hidden groups in data, using patterns of co-occurrences. Such patterns cannot easily be detected by conventional approaches, let alone us as researchers may not be aware of such associations in their data [32, 39]. Noteworthy, is that the LDA approach often struggles to capture the intricate nuances of language and context, and its implementation requires some programming expertise [10, 40].

#### Topic modelling using BERTopic

BERT, being a contextualized language model, takes into account the surrounding words when representing each word in a sentence. This bidirectional understanding enables BERT to create more accurate and contextually rich word embeddings. In the context of topic modelling, these embeddings can be utilized to derive topic representations that better reflect the semantic relationships between words [40].

The work of Scarpino et al. [41] supports this notion, as they report that BERTopic outperforms LDA approach in clustering in their study that investigated topic modelling techniques to extract meaningful insights in Italian long COVID narrations. The study of Silva et al. [42], reports on the successful application of BERTopic approach as an unsupervised natural language processing in the identification of patients with suspected COVID-19 infection. Similar successful application of BERTopic as a topic modelling approach in various health spaces is reported in [43–49]. As such, the application of BERT in topic modelling holds the promise of enhancing the sophistication and contextual relevance of topic extraction, thereby contributing to more accurate and meaningful insights derived from textual data.

The text mining process entailed the following steps; data acquisition, pre-processing, feature extraction, topic segmentation, and discovered knowledge. These techniques are reported elsewhere [11, 12, 15, 24, 36–38, 50].

## Materials and methods

In this study, we apply TM techniques to extract mortality causes and identify the most prevalent diseases using VA data. This is a retrospective cross-sectional study that uses secondary data analysis through TM methods. The VA data was obtained from the study area of the Agincourt Health and Demographic Surveillance System (HDSS). This study has been approved by the relevant ethics committee in South Africa (University of the Witwatersrand Faculty of Health Sciences, Human Research Ethics Committee (Medical), and (approval ref. no: M1911132). It was conducted in accordance to the Declaration of Helsinki. Informed participant consent was waived by University of the Witwatersrand Faculty of Health Sciences, Human Research Ethics Committee (Medical). We got permission from the Agincourt Health and Demographic Surveillance Site to use their data for research purposes from 29 November 2019 to 29 November 2024. The inclusion criteria was all VA autopsy data for the period between 1993 and 2015.

The HDSS was established in 1992 and is situated in the rural Sub-district of Bushbuckridge under Ehlanzeni District, in Mpumalanga Province, in north-eastern South Africa. The geographical coverage is approximately 420km^2^. As of 2019, the population was at 116 247 individuals residing in 28 villages with 22 716 households, with males being 55 961, females being 60 280, children under 5 years being 11 724 and school going children with ages from 5–19 being 35 928 [51].

The Agincourt HDSS is a surveillance site that serves as an evidence based health monitoring site that seeks to strengthens health priorities, practice and inform policy. The data was collected between 1993 and 2015. For this study, we only use textual data as VA narratives denoting one feature with 16338 cases / observations, which where in English. However, for our semantic and structure analysis we compare our extracted symptoms from the VA narratives to that of the responses from the structured questionnaire. The responses from the questionnaire dataset entailed 227 symptoms. All symptoms that had a ‘Y’ were encoded as a 1 meaning that the record had a present symptom value. On the other hand, all features that had a ‘N’, were encoded as a 0 meaning that those records had no symptoms present. All our features were in English. This comparative analysis was done to investigate if mined narratives exhibit similar and/or have additional features.

**Fig 1:**
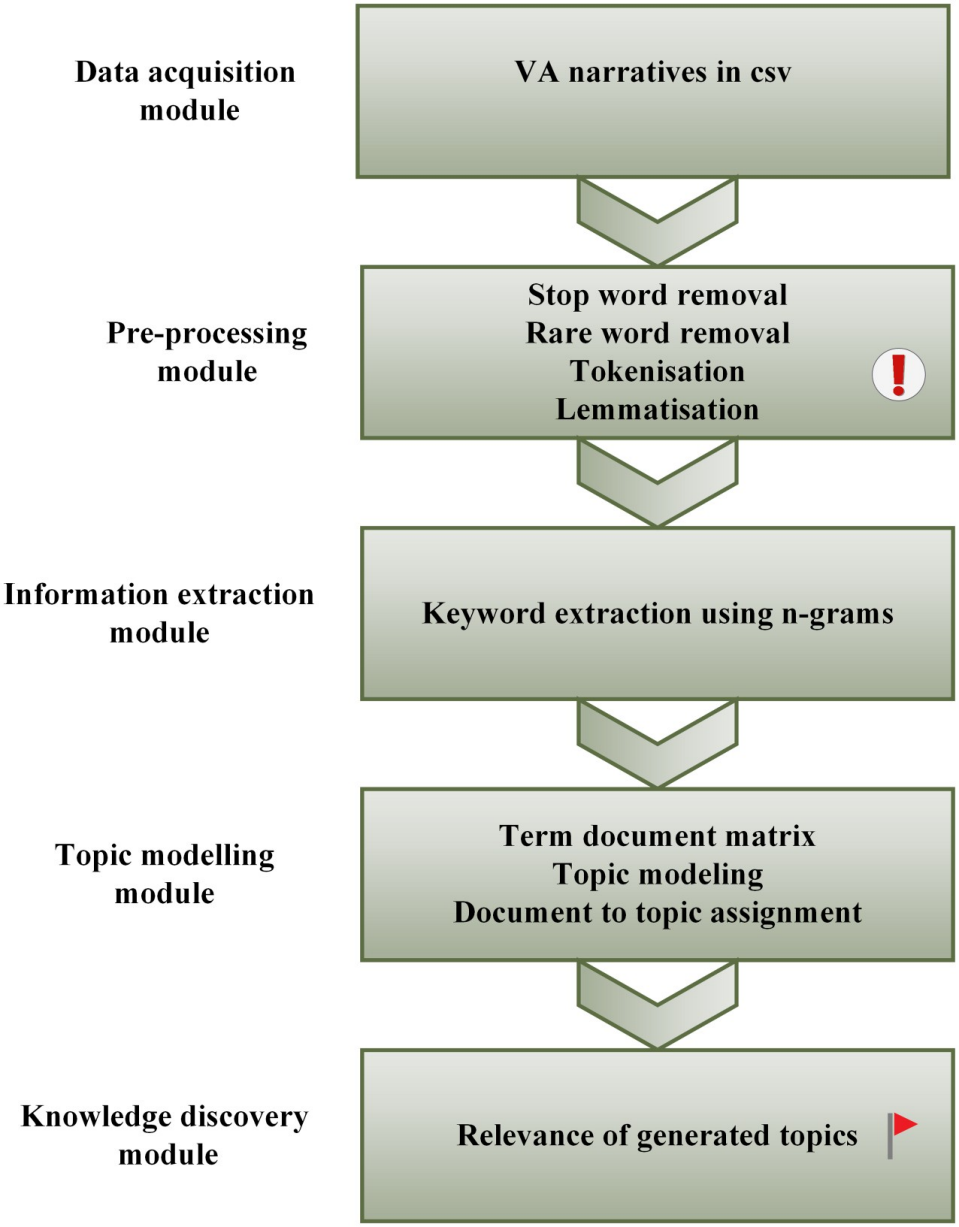
Text mining steps followed in this study.

In this study, we first do data acquisition of our VA narratives as a comma separated value text file (csv), followed by preprocessing, then keyword the extraction using n-gram natural language processing, followed by topic modelling using LDA and BERTopic and lastly identifying the relevant topics through knowledge discovery. We follow the TM steps as depicted in Fig 1 below.

### Data pre-processing

We performed pre-processing as part of data cleaning and normalisation, as we sought to do away with irrelevant data. In most cases input of textual data requires pre-processing and transformation before being mined [52]. After data acquisition, in csv format, we started by cleaning the unstructured narrative data by converting all text to lowercase, removed all punctuation, spaces, numbers and special characters. We applied stopword removal in order to remove insignificant words using the NLTK library of English stopwords. We performed tokenisation which is breaking up of text into tokens, of words or sentences. Lemmatization was applied by employing the Python Spacy package. This process uses a dictionary of known word forms and considers the role of a word in a sentence with the aim of extracting some normal form of a word (See Fig 1, Pre-processing module).

### Information extraction

After the pre-processing phase, we now had our tokens as words, and at this stage features could now be defined. The features were extracted as tokens of words, using n-gram natural language processing. The n-grams were seen as tokens of adjacent words denoted as; *n* = 1 a unigram such as, *‘pain’*, *n* = 2 a bigram, such as *‘chest pain’*, *n* = 3 a trigram, such as *‘high blood pressure’* and *n* = 4 a quodgram, such as *‘blood result hiv positive’* [31]. The bigger the n-gram word frequencies, the more contextual information that we extracted. These n-grams models gave us an understanding of our narrative corpus and denoted the frequently occurring terms, also known as symptoms or mortality causes (See Fig 1, Information extraction module).

Additionally, we also performed keyword extraction defined as a representation of a document’s content using a word or a sequence of words. These words provide meaning within a sentence’s context. As such, one can discover hidden patterns and correlations using TM approaches in an automated fashion from large volumes of unstructured data using keywords [7]. Recent novelties in TM, now allow the extraction of keywords from documents without the assistance of experts, a process known as expert driven.

Keyword extraction is a form of text analysis that seeks to pull out relevant keywords automatically from unstructured text. These keywords are a good descriptive summary of the unstructured text. In this study we use Term Frequency with Inverse Document Frequency (TF-IDF). This approach mainly captures the most frequently occurring word(s) in one document and less less frequently available in other documents [7] (See Fig 1, Information extraction module).

### Topic modelling using LDA

The LDA components that we used entail; *a) Term document matrix*, that represents a document as a bag of words. It is also a easier way of representing the corpus, known as a word document matrix. The word document matrix is then taken as input and fed in topic modelling [5, 53], *b)Topic modelling* using LDA. This approach is statistical in nature and is used for clustering text documents. LDA makes the assumption that each document is made up several topics and on the other hand each topic has various words belonging to it. The representations are done through document-topic *w* distributions and topic-word distribution *v*. As such, *w* and *v* are considered as dirichlet distributions. In the process the topics are discovered since they are not predefined, and *c) Document to topic assignment* is the most useful process of the LDA algorithm [53].

#### LDA algorithm

The LDA process follows an iterative manner starting with a random sample. This implies that the LDA process seeks to maximise the probability of a document belonging to a particular topic, and the probability of a word belonging to a certain topic iteratively. Once the algorithm converges, it generates final collection of words that are representative of a particular topic. It then computes the topics based on probability, and the topic with the highest weighting will denote the document’s most dominant topic (See Fig 1, Topic modelling module). It is key to find an optimal value relative to the topic distribution per document using the hyper-parameter *α*. A high value of *α* may lead to a homogeneous distribution of topics. On the contrary, a low value of *α* may hinder a fair topical percentage distribution and any inferences thereof [53]. The LDA approach we use is reported in [7, 10, 53].

**Fig 2:**
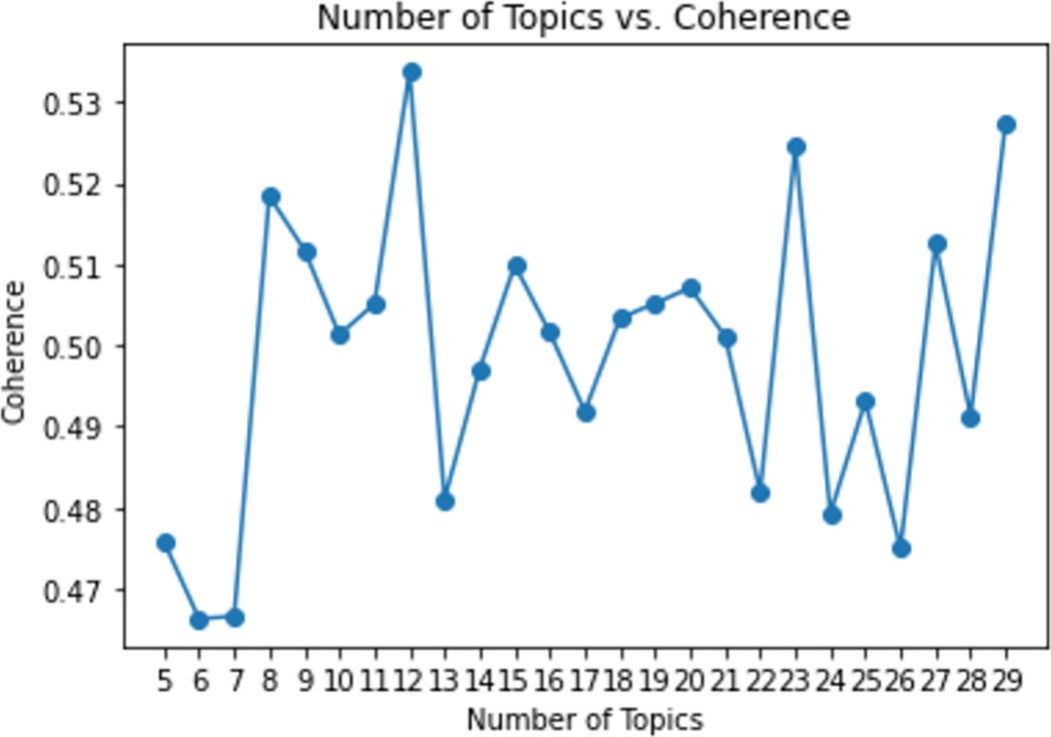
Hyper-parameter optimization for number of topics.

We applied the pyLDAvis package in Python in order to discover important information about our topics. We used a coherence score of 19 topics which we derived from the exhaustive search of the optimal number of topics and *α* (See Fig 2). The inter topical distance graph depicts topics as circles, and the distance between the circles denotes the relationship between topics (See Fig 6). The relationship is generated through dimensionality reduction using principal component analysis which employs probability distributions. The goal of this process is to try and have optimal topics that are distinct and do not overlap. To achieve minimal overlap we have to optimise our model by fine tuning model parameters such as *α* and relevancy (method for ranking terms within topics) to attain terms that are most important and exclusively belong to a particular topic. This overcomes the issue of having a biased representation of a topic where highly ranked terms maybe frequent across the whole corpus [54]. In this study we follow the LDA methodological algorithm and approach reported in [53].

### Topic modelling using BERTopic

This present study implemented BERTopic in four steps namely; 1) converting documents to their embedding representation using a pre-trained language model, 2) using uniform manifold approximation and projection (UMAP) for dimensionality reduction, 3) applying the hierarchical density-based spatial clustering of applications with noise (HDBSCAN) algorithm to cluster VA narratives into categories that have a similar meaning, and 4) generating classes through a class-based term frequency with inverse document frequency (c-TF-IDF) weighting scheme based on importance and relevance of a word, thus denoting the most representative words for each topic [41].

#### BERTopic algorithm

We used the BERTopic library in Python in order to discover important information about our topics. The BERtopic algorithm starts by creating document embeddings. Documents are embedded to create representations in a vector form that is semantically related. This implies that documents that entail the same topic are semantically similar. Document embedding is done using sentence-BERT framework, thus enabling users to convert text in sentences and paragraphs to dense vector representations using pre-trained language models. As such, these embeddings can be applied to perform clustering of similar documents. However, the embeddings are not used in generating topics [55]. After creating the document embeddings, the algorithm then performs dimensionality reduction using UMAP. This results in a more representative vector space which preserves local and global features. The reduced document embeddings are then clustered into similar groups using HDBSCAN. During the clustering process, HDBSCAN also clusters noise as outliers. Ultimately, this avoids clustering unrelated documents and improves the process of topic assignment. Lastly, topic assignments are done using c-TF-IDF, and are based on the documents in a cluster, where one topic will be a cluster [40, 45, 55, 56].

#### Optimisation of LDA and BERTopic algorithms

We carried out exploratory topic modelling through an exhaustive search of the number of topics. In order to attain an optimal number of topics *k* we fine tuned *α* our hyper-parameter for the LDA algorithm. Additionally, we explored with number of topics from *k* = 5 to *k* = 30, and we looked at values of *α* that had a uniform distribution with values in the range 0−1, and these where (0.49, 0.504, 0.505, 0.508, 0.50, 0.48). After experimenting with (*k* = 12, *k* = 19, *k* = 23, *k* = 29) topics, we discovered most coherent topics using 19 topics, as most of the words in a topic were associated. It should be noted that even if we generated high coherence scores for (*k* = 12, *α* = 0.54), (*k* = 23, *α* = 0.525), and (*k* = 29, *α* = 0.53), we settled for (*k* = 19, *α* = 0.505), since an increase in topic numbers results in difficulties in terms of human interpretability. Moreover, a large *α* value leads to similar generation of words in topics. Therefore, we chose lower values that follow a normal distribution and had most diverse representative terms [53]. Fig 2 shows the exhaustive search process that was done to identify the optimal number of topics.

The implementation of the BERTopic algorithm entailed optimisation through the use of UMAP to improve topic assignment. BERTopic model evaluation was done using c_v (it is based on a sliding window, one-set segmentation of the top words and an indirect confirmation measure that uses normalized pointwise mutual information (NPMI) and the cosine similarity), and c-umass (it takes into consideration the document co-occurrence counts, one-preceding segmentation, and a logarithmic conditional probability as a confirmation measure) [57]. Both measures seek to determine the degree of significance of words in a topic and the level of interpretability, thus assessing topic quality from a human perspective. The c_v measure ranges between 0 and 1. The higher the c_v score, the more understandable and coherent a topic is to a human. On the contrary, the closer the c-umass score to 0 the better [57]. The BERTopic attained a c_v of 0.66 and c-umass of −0.32. The mathematical approach is reported in the study of [40, 48].

### Knowledge discovery

Zhu et al. [24] defines knowledge discovery as identifying meaningful information from large volumes of unstructured text. This knowledge can entail implicit or explicit facts, information or descriptions which relates to the contextual understanding of a specific domain. Furthermore, this discovered knowledge can serve as extra information or data that can be further utilised to discover more interesting patterns. In our context we employ knowledge discovery through data mining of the VA narratives in order to find answers to mortality causes and the most prevalent diseases that might have attributed to high mortality numbers within the HDSS. Additionally, further deductions and inferences through integration of our biomedical VA narrative data with other sources of data can give us further insights on our investigations (See Fig 1, Knowledge discovery module).

Buenano-Fernandez et al. [53] points out that at this phase we have to identify relevant topics by adding topic labels through a synthesis of the content generated by the topic assignment algorithms. However, the LDA approach has a challenge of interpretability, as the generated topics are not easily interpretable by humans because the initial assignment of words is random. Therefore, it is of paramount importance to have human interaction from beginning to the end of the process, in order to attain an optimal final model. As such, we engaged with a subject matter expert to assist with the topic labels. The expert synthesised the generated tokens and allocated a probable disease category. Similar steps and consultations were done with experts in order to get the best probable cause of death on the BERTopic algorithm results.

## Data analysis

In this study we performed information extraction through word frequencies (n-grams) and keyword extraction using TF-IDF to identify the most frequent terms in the corpus, for our data analysis phase. We removed 2247 cases that had missing textual narratives. We further chucked out terms that had a high and low sparsity respectively, thus they were infrequently occurring in the corpus. We ended up with a 14079 documents for our analysis. Additionally, we also employed inter-topic distance measure through principal component analysis and applying HBSCAN to understand how our topics are related and clustered in our corpus. Moreover, we also performed structure and semantic analysis of random twenty (20) narratives in order to investigate if the narratives have same or additional variables as compared to the symptoms/variables from the responses to the structured questionnaire.

## Statistical packages

All statistical analyses were done using Python 3.11.5 using Jupyter Notebook platform. The following libraries were used; *pandas* and *numpy* for data manipulation and analysis, *nltk* for preprocessing, *matplotlib* for generating visualisation, *sklearn* for feature extraction *bertopic*, *gensim* and *spacy* for topic modelling.

## Results

The extracts below describe the results attained from the n-gram natural language processing approach and topic modelling.

### Term frequencies using word cloud

**Fig 3:**
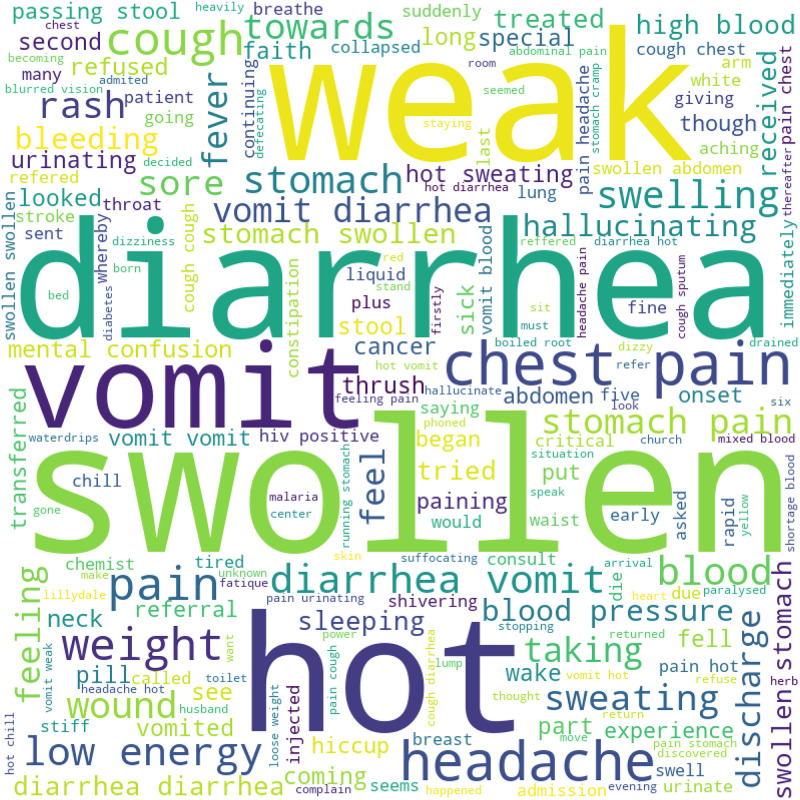
Word frequencies using word cloud.

Fig 3 depicts the the word cloud of terms that we extracted from our VA corpus. The word cloud captures a visual illustration and representation of word frequency. The more bold and large the text is, the more important it is.

### Term frequencies using TF-IDF

Table 1 depicts the top 8 word frequencies using TF-IDF, a process known as keyword extraction. We found out that terms like *swollen, pain, hot, weak, diarrhoea, headache, stomach, vomiting, swollen, stomach, mental confusion, hiv positive, high blood pressure, cough, and chest pain* had high weightings in that listing order signifying their importance in corpus. Additionally, terms like *chest pain, low energy, diarrhoea vomiting* also had high weightings in the corpus. This suggests that these terms had high frequency scores, denoting importance in a document but less common in other collections of documents. Similarly, we can deduce that these term’s importance was high in given documents, but very rare in other documents.

**Table 1:** Most frequently occurring terms.

Term	TF-IDF
pain	0.0926
swollen	0.0925
weak	0.0763
diarrhoea	0.066
vomit	0.058
hot	0.057
cough	0.055
chest pain	0.053

### Term frequencies using n-grams

**Fig 4:**
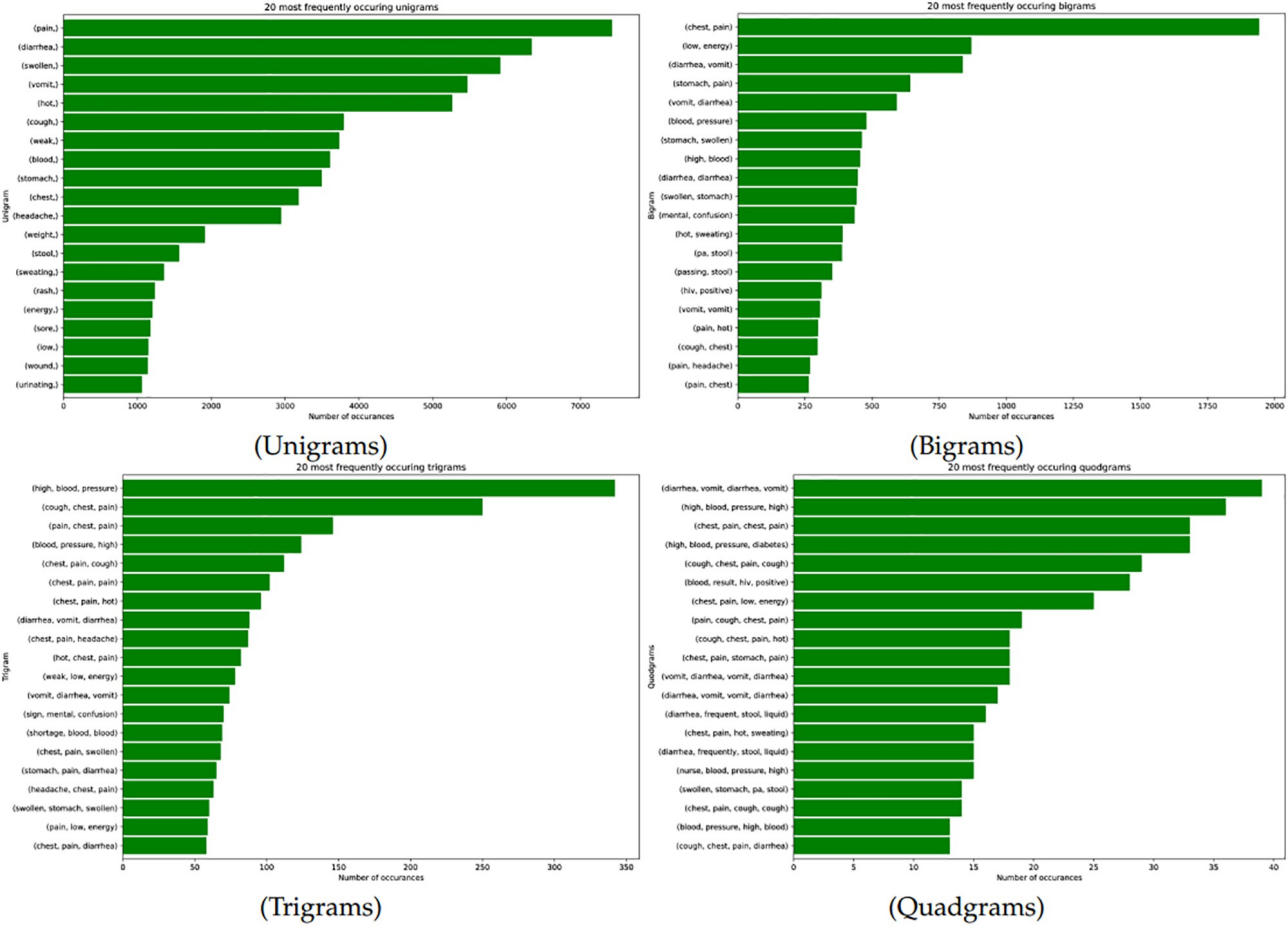
Most prevalent mortality causes using n-grams language processing.

We illustrate our word frequencies in Fig 4 using n-grams. Firstly, we depict a visual representation of the most common symptoms from our corpus using uni-grams. In a similar fashion, we managed to discover the same frequent terms as in our TF-IDF approach and the word cloud. We can see that the symptoms such as *pain, diarrhoea, swollen, hot, cough, weak, blood, stomach, chest, headache and vomiting* formed part of the most occurring uni-grams. Moreover, from our bi-grams, we can see that *(chest, pain), (mental, confusion), (swollen, stomach), (diarrhoea, vomiting), (diarrhoea, diarrhoea), (high, blood),(stomach, swollen),(blood, pressure),(stomach, pain) and (low, energy)* were the most frequently occurring symptoms. Tri-grams show that *(chest, pain, cough), (blood, pressure, high), (pain, chest, pain), (cough, chest, pain) and (high, blood, pressure)* were the most occurring symptoms. The quad-grams show that, *(diarrhoea, vomit, diarrhoea, vomit),(vomit, diarrhoea, vomit, diarrhoea), (chest, pain, stomach, pain), (cough, chest, pain, hot), (pain, cough, chest, pain), (chest, pain, low, energy), (blood, result, hiv, positive), (cough, chest, pain, cough), (high, blood, pressure, diabetes), (chest, pain, chest, pain), (high, blood, pressure, high)* as the most occurring symptoms. Based on these word frequencies, one can possibly deduce that, these symptoms were the most common mortality causes of our HDSS population.

### Semantic and structure analysis of additional and similar variables

**Fig 5:**
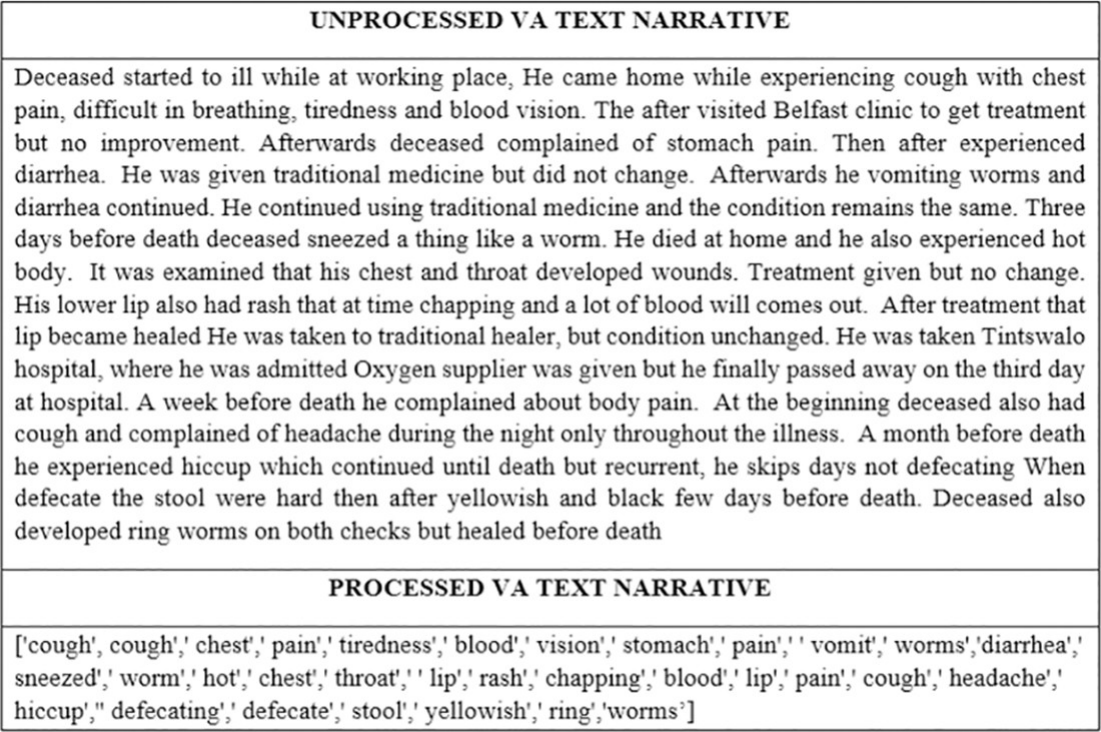
An example of a processed VA narrative.

This section discusses results of the structure and semantics analysis of twenty random cases that we extracted to investigate if the narratives had additional information or exhibit the same symptoms as in the responses from the structured questionnaire. Interestingly, we note that the narratives entail the same symptoms as in the responses from the structured questionnaire, and further analyses gave us insights that they also entail additional variables that can be used to help with disease diagnosis especially in cases where the cause of death is unknown or there is a disagreement between physicians. Table 2 below is a comparison of symptoms from the responses from the structured questionnaire and symptoms the extracted textual data. Symptoms highlighted in the colour yellow denote similar symptoms and orange colour additional symptoms. We used anonymous record identifiers. Fig 5 is a VA narrative example that shows how text was preprocessed to generate clean processed symptoms/tokens that were used for the comparative analysis with responses from the structured questionnaire.

**Table 2:** Comparison of symptoms from textual data and responses from structured questionnaire.

RecID: Symptoms from questionnaire	Extracted symptoms from textual data
A1: fever, cough, breath, chest pain, whooping	cough , whooping, cough, screaming, vomited, hot, swollen
A2: cancer, fever, sweating, breath low, yellow, abdomen pain, urine, hair, weight loss	stomach, vaginalbleeding, womb, cancer treatment, painful,stomach bleeding, urinating, swollen breast
A3: hypertension,asthma,low breath, weight loss, swollen legs, alcohol intake	passingstools , swollen
A4: hypertension, skin, urine, weight loss, swollen legs, excessive drinking	develop, soretoe, toesinfection, wounds, grow infection, smell, worms, swollen
A5: fever, breath, chest pain, weight loss, excessive drinking, sweating	carrying , heavybox, twisted, pains, toes, affected, toes, swell, pain, blood, clot, removed, swell, huge, lumpbursts, holeslump, chest, armpits, groin, throat, lumps, busted, pusscoming
A6: TB, epilepsy, fever, cough, breath low, chest pain, diarrhoea, convulsion, vomiting, stiff neck	swollen , diarrhoea, stools, cough, go, toilet
A7: nil	pain , treated, looking better, weak, admitted, drips injection, worse
A8: hiv, fever, cough, low breath, yellow, alcohol, diarrhoea, vomit, weightloss, abdomen, swelling	sweats , loseweight, vomit, weak, hiv, water drips, diarrhoea, hallucinating, swollen stomach
A9: sweating, cough, low breath, urine, weight loss, paralysed, alcohol	cough , heavily, rot, worms, rotting, spread, thigh, buttocks, waist, extent, intestines, visible, abdomen
A10: fever, cough, vomit, weight loss, alcohol	cough , signs, vomits, low, energy, coughcritical, tried, natural, fever
A11: hypertension, fever, low breath	fell , forehead, improve, speak, tears, rolledeyes, mixedblood, accident, urinate
A12: fever, cough, low breath, abdomen, swelling, headache, injury	initially, swollenears, swollen, abdomen, critical, puss, ears, fever, cough
A13: nil	swollen , wounds, top, feet, wounds, burst, puss, swollen, feet, cuts, razorblade, rubbed
A14: fever, low breath, chest pain, diarrhoea, vomit, weightloss, hair,eyes sunken	committing, thinner, diarrhoea, oral, dehydration, solution, vomit
A15: chronic, fever, skin, weight loss, swollenlegs, alcohol, smoking	pain , toe, long, kneel, cut, fleshwound, toe, swollentoe, infected
A16: fever, cough, diarrhoea, blood diarrhoea, vomit, weight loss	diarrhoea , vomit, weightloss, oral, dehydration, thrush
A17: hiv, stroke, low breath, weight loss, alcohol, chest pain	fell , weak, touch, hold, feeling, pains, arv, years, clear, pass, stools, giving, arv, sweats, minutes

We can see that from Table 2, we managed to elicit similar symptoms from the textual data as compared to the responses from the structured questionnaire (see A1, A2, A8, A10, A16, A17). We also note that the extracted textual narratives also exhibit additional symptoms that can be easily interpreted to assist physicians in reaching a diagnosis (A5, A8, A9, A11, A14, A15, A17). On average from our random sample of cases, the retrieval rate of additional is approximately *n* > 2, per every case where *n* is the number of terms. Moreover, we discovered that the text-extraction algorithm misses a symptom that is in the responses from the questionnaire cases ≈2% of the times. This can be attributed to the fact that some of the narratives entail shallow content and are less informative than others.

### Topic modelling results using LDA approach

**Fig 6:**
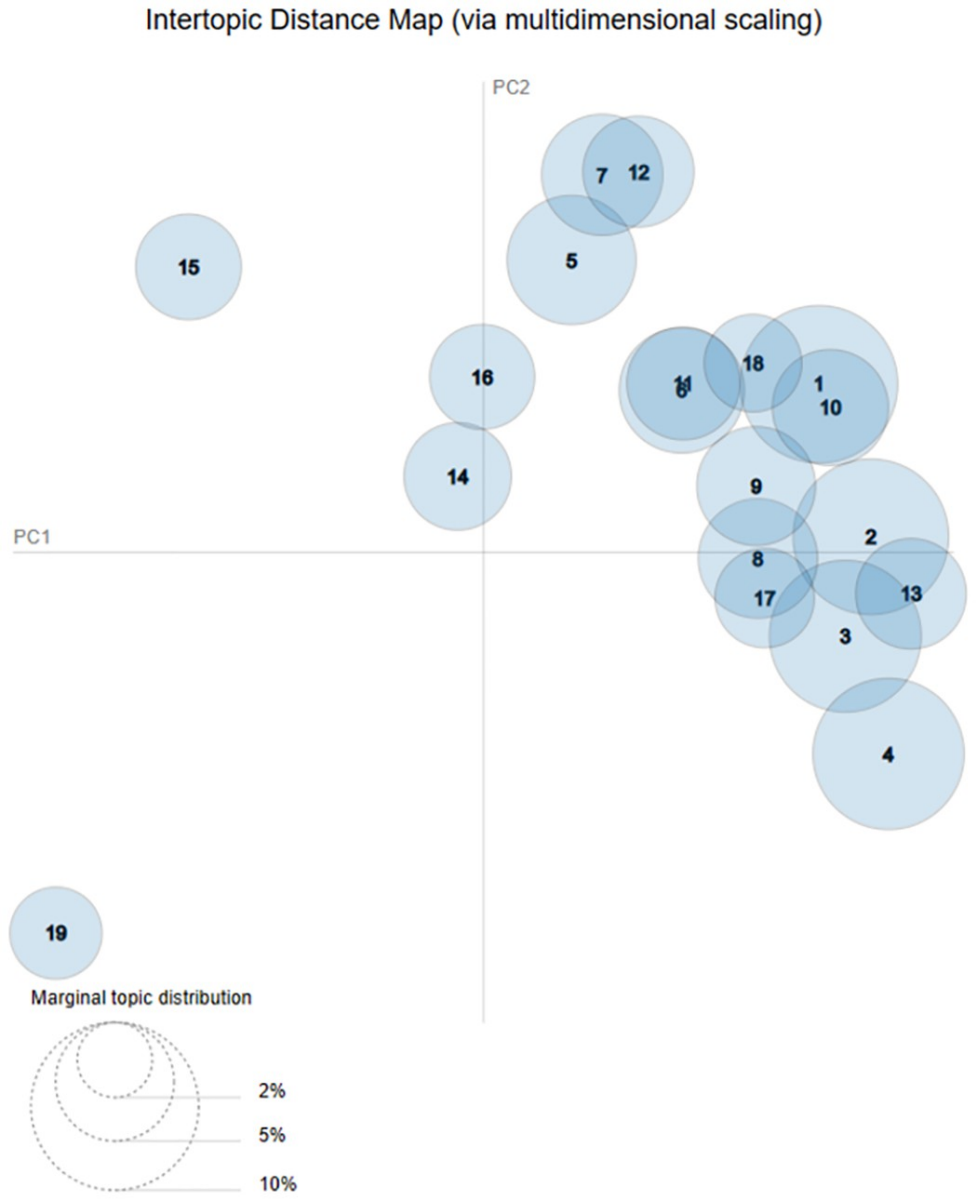
LDA inter-topic distance map using multi-dimensional scaling.

In this section we illustrate through graphs the results of our LDA process. Table 3 shows the results of the common 19 topics that we mined from our VA narratives (the most prevalent diseases). Additional visualisation is also given on the inter topic distance map in Fig 6. Basically, we noted that the most prevalent diseases were mainly communicable and non communicable. The highlights being *diarrhoea, TB, HIV, malaria, chronic ailments, neurological disorders, asthma, accidents and maternal related deaths*. We also had topic 7 and 12 which had fewer symptoms to relate to any disease and we categorised them as unknown. Expectedly, we note that maternal and accident related deaths which are topic 19 and 15 respectively are distinct and far from the other clusters. This is attributed to the fact that they have very dissimilar symptoms from the other topics. We also discovered that topic 14 and 16 which are mental confusion and heart ailment respectively, are on their own cluster. This suggests that the two topics share some common similar symptoms. Another interesting observation is the cluster with 5, 7, and 12 which are topics for chronic liver ailments and unknown topics respectively. This grouping depicts similar symptoms for unknown which are also present in the liver complications. This suggests that cases with such symptoms in the unknown can have symptoms that lead to liver complications.

**Table 3:** Most prevalent diseases identified using LDA.

Topic number and name	Tokens
T1: Diarrhoea/respiratory tract infection	cough body diarrhoea weight hot sputum rapidpains vomit headache rash chills
T2: HIV	diarrhoea vomit hiv hot weak positive refuse arv blood cdcount stopped
T3: Tuberculosis	cough pain chest sputum hot lungs weight results sharp spitblood lungs
T4: Cervical cancer	bleeding blood cancer urinate swollen urine wound urinating coming vaginal nasal puss
T5:Chronic liver ailment	paining stools pass pains liver weak running constipation stomach admitted shortness tired
T6:Neurological disorders	diarrhoea looked mental confusion hence vomit weight encountered abscess suddenly improve though
T7:Unknown	pains chest headache feelinghot hallucinating abdominal migrant faith breathe energylow
T8: Breast cancer/infection	wound swelling swollen feet sore puss lump burst ointment wounds breast sent hole hip infected
T9: Chronic ailments (liver,lung, kidneys)	swollen abdomen liver discharge diarrhoea vomit yellow drained lungs kidneys lungs swell
T10: Malaria	received malaria breast oral groaning feeding diarrhoea vomit toilet problems dizziness dehydration
T11: Jaundice/malaria/skin infection	rash sores vomit itchy yellow diarrhoea thrush hoteyessunken weak sweating
T12: Unknown	panado syrup lillydale years liquid problems toilet die go oral whenever going
T13: Diabetes/Hypertension	high blood pressure stroke collapsed diabetes low hot energy shivering paralysed hours
T14: Mental confusion	feels starts refuse injected admission confused weak hiccups care mental makes sick
T15: Accidents	car accident shot road broken truck driving taxi fell collapsed paralyzed hanged
T16: Septic wound/heart ailment	wound received swelling began whilst swollen stomach cramps herbs hot bigger sore
T17: Epilepsy	stiff tongue teeth bent gone onset epilepsy jaws pills uncontrolled neck sudden
T18: Chronic ailments (heart)	heart fast swelling failure beating collapse urinate pass penis catheter bladder paralysis
T19: Maternal and Asthma	crying hours born room pregnant breast delivered delivery asthma breastfeeding cried incubator

One interesting discovery is that topic 2 on HIV is central to most of the clusters such as TB, diarrhoea, malaria, cancer, epilepsy, diabetes, hypertension, and chronic ailments. One possible reason for this is that most of these disease are related to HIV and most cases who have these complicated diseases also have HIV, or might be at risk of having HIV, as evidenced by the similar symptoms. Furthermore, we also observe that jaundice/ malaria with skin infection is related to neurological disorders as evidenced by the close cluster embedding of topic 6 in 11. This suggests that some cases had a long untreated jaundice which might have led to brain damage, a condition known as kernicterus. Of particular interest is cluster diarrhoea with respiratory tract infections, malaria and chronic heart ailments (topic 1, 10 and 18). This suggests that these diseases share similar symptoms and if one suffers from the earlier diseases, it can lead to heart chronic ailments. Another finding is that cluster 8 and 9 on breast cancer and chronic ailments respectively have similar characteristic features. This suggests that if one has cancer they might also have complications of liver, lungs, and kidneys.

Fig 6 depicts the relationship and association between topics using the inter topic distance measure through principal component analysis. This process basically illustrates and models the visual representation of similar topics being in close proximity (one cluster) and dissimilar topics distant to each other. The bigger the topic circle the more tokens it uses from the corpus for topic generation. As such, we can see that topic 1, topic 2, topic 3 and topic 4 use 8.8%, 8.6%, 8.2% and 8.1% of the tokens in the corpus respectively. Topics that are overlapping imply having some similar characteristics symptom wise, hence they fall within the same cluster. We can see that topic *15* and *19* are distinct topics. Noticeably, we can also see clusters *(14 and 16)*, *(5, 7, 12)*, *(3,4)*, *(2, 3, 13)*,*(9,8,17)*, *(11,6)*, *(18,1,10)* having similar characteristics.

### Topic modelling results using BERTopic approach

**Fig 7:**
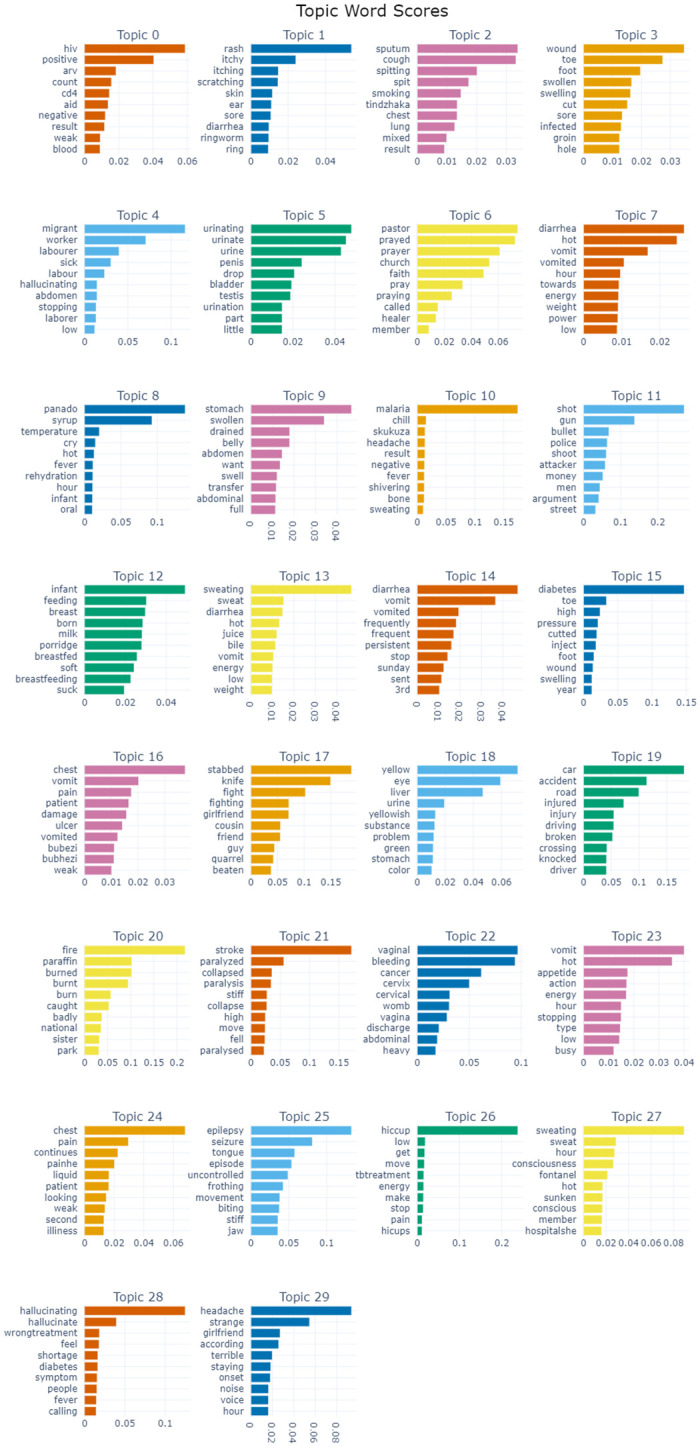
Topics generated using BERTopic model.

This extract uses figures to depict the results of our BERTopic model in topic assignment. Initially, the model generated 154 topics, identified 6265 documents as outliers. UMAP was applied for dimensionality reduction and we were left with 30 topics. Fig 7 shows the generated thirty topics and corresponding top words with scores for each topic. Topic 0 is about HIV that has top word scores of *hiv, positive, arv, count*. Topic 1 shows skin related diseases. Topic 2 is TB as evidenced by symptoms with high word scores such as *sputum, cough, smoking, chest, lung*. Topic 3 suggests septic wound diseases which might be attributed to other diseases such as diabetes. Topic 4 is unspecified. Topic 5 is a sexually transmitted diseases as evidenced by the genital related symptoms. Topics 6 and 8 are unspecified. Topic 7 and 14 denotes symptoms that might be related to diarrhoea. Topic 9 is about abdominal swelling complications. Topic 10 is about malaria. Topics 11 *shot, gun, bullet, police, shooter*, 17 *stabbed, knife, fight*, 19 *car, accident, road, injured, driving*, and 20 *fire, paraffin, burned, burnt* are accident related causes of death. Topic 12 is maternal related death as highlighted by these high word weightings *infant, feeding, breast, born, milk*. Topics 13 shows symptoms that might be related to other diseases such as HIV. Topic 15 suggests diabetes and hypertension related causes of death as denoted by these terms *diabetes, toe, high, pressure, cut*. Topic 16 suggests ulcers and TB related symptoms. Topic 18 might be related chronic liver ailments and jaundice like disease. This is shown by high word terms like *yellow, eye, liver, urine, yellowish, substance*. Topic 21 is related to stroke and paralysis possibly because of diabetes and hypertension causes, with terms such as *stroke, paralysed, collapsed, paralysis, stiff*. Topic 22 talks to cervical cancer prone in women as shown by these terms *vaginal, bleeding, cancer, cervix, cervical*. Topic 23 shows symptoms that are related to diseases such as malaria and HIV. Topic 24 depicts symptoms that are TB related. Topic 25 is a result of epilepsy evidenced by top word scores such as *epilepsy, seizure, tongue, uncontrolled, stiff, jaw*. Topic 26 exhibits hiccup symptoms coupled with pain and low energy. This suggests possible diseases such as pneumonia, bowel diseases, pancreatitis, pregnancy, bladder irritation, liver cancer or hepatitis. Topic 27 show symptoms related to diabetes such as consciousness, sweating, hot body and sunken eyes. Topic 28 is related to psychiatric conditions because of high weighting on terms like hallucination. Topic 29 is unknown.

**Fig 8:**
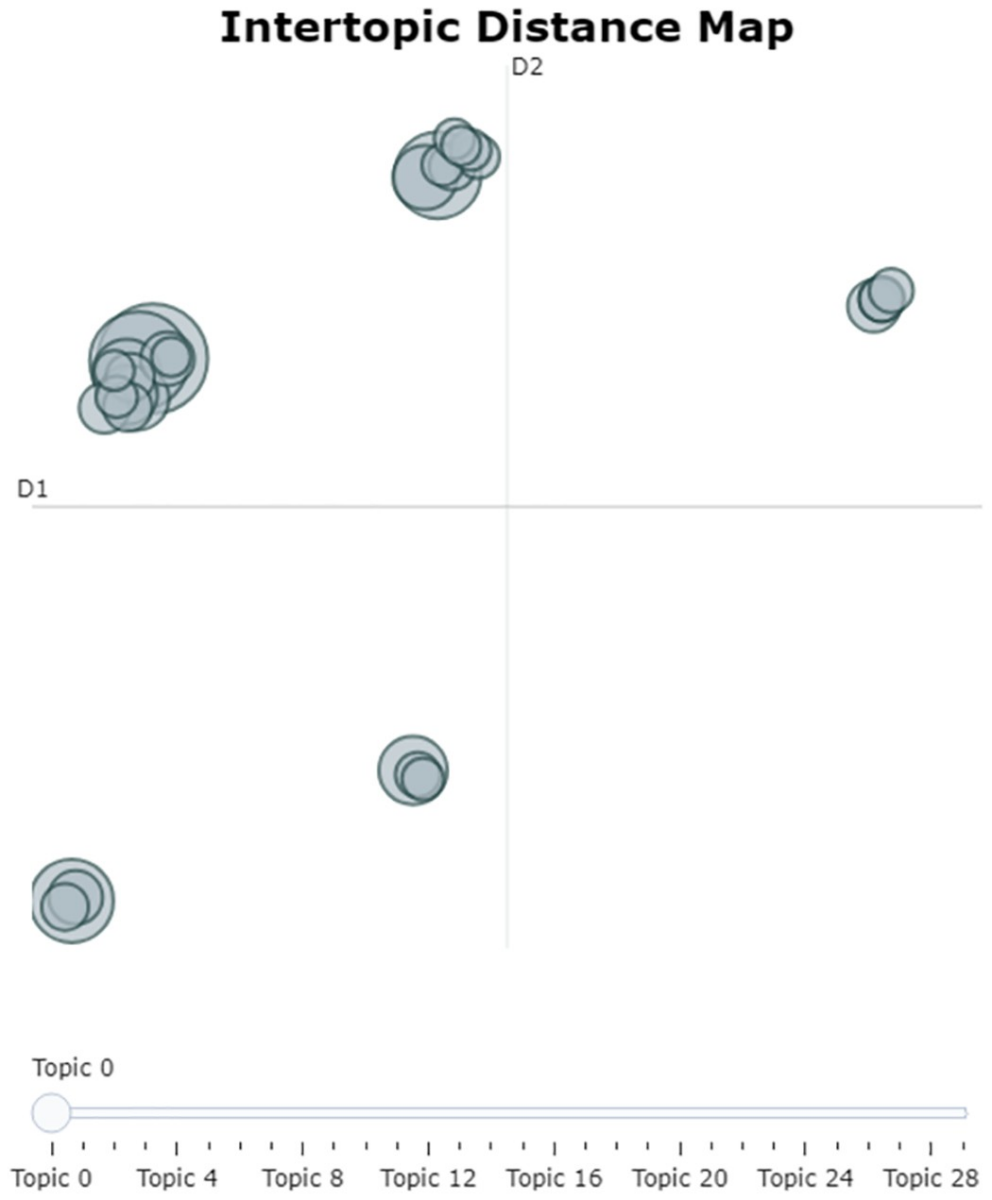
BERTopic inter-topic distance map.

Fig 8 shows the inter-topic distance map with 5 dominant clusters. Cluster 1 is made up of topics (5, 18, 22). These are correlated due to genital mortality causes both in men and women. This cluster suggests sexually transmitted diseases and infections that ultimately lead to liver and chronic ailments. Cluster 2 is made up of topics (3, 9, 15). This cluster denotes diabetes and high blood pressure causes of death. These chronic diseases can lead to amputation and abdominal complications. Cluster 3 shows correlation of topics (11, 19, 20) that are accident and injury related causes of death. Cluster 4 shows the dominant HIV as topic 0 and closely correlated to topics (1, 4, 7, 8, 12, 13, 14, 23, 27, 29). This suggests that most of the HIV diseases are related to diarrhoea, and symptoms such as *low energy, no appetite, headaches, skin infections, vomiting, loss of weight, sweating*. Interestingly, we observe that similar symptoms are correlated to maternal deaths. Noteworthy, is the striking number of migrant workers who exhibit same symptoms and also succumbed to death from HIV and maternal related diseases. Cluster 5 is made up of topics (2, 6, 16, 21, 24, 26, 28). Topic 2 which is related to TB and lung diseases is closely correlated to topic 16 that talks to chest pains. Noteworthy, we observe epileptic seizures being correlated to loss of consciousness, paralysis and collapsing.

**Fig 9:**
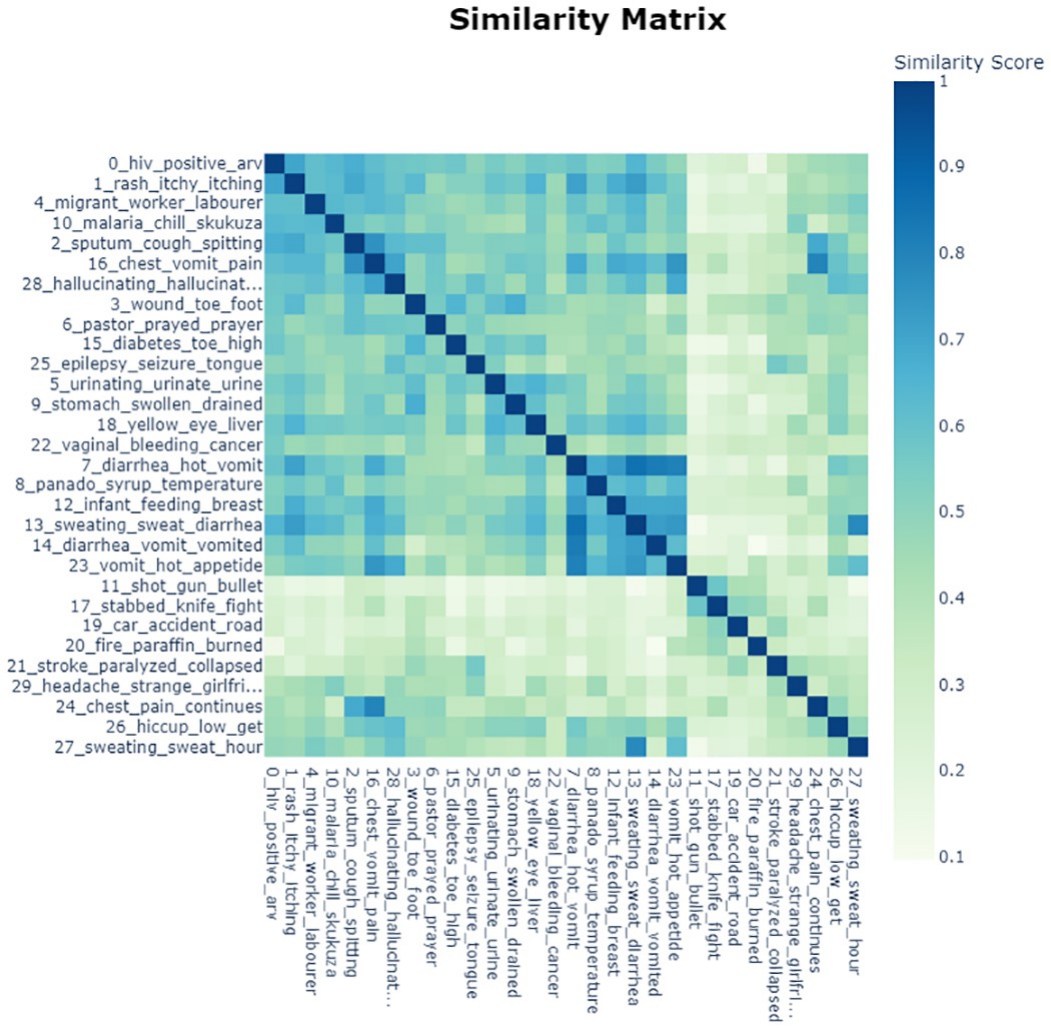
Heatmap similarity matrix.

Fig 9 shows a heatmap similarity matrix with distinct topics, generated from BERTopic document embeddings. This was a result of applying the cosine similarity to document embeddings to get the relationship and distinction between topics in a quantifiable way. Noteworthy, the similarity score on the topics on HIV, skin related diseases, migrant workers, malaria, TB, chest pain, hallucination, septic wounds, diabetes and hypertension, and epilepsy are very high amongst others as depicted in Fig 9.

**Fig 10:**
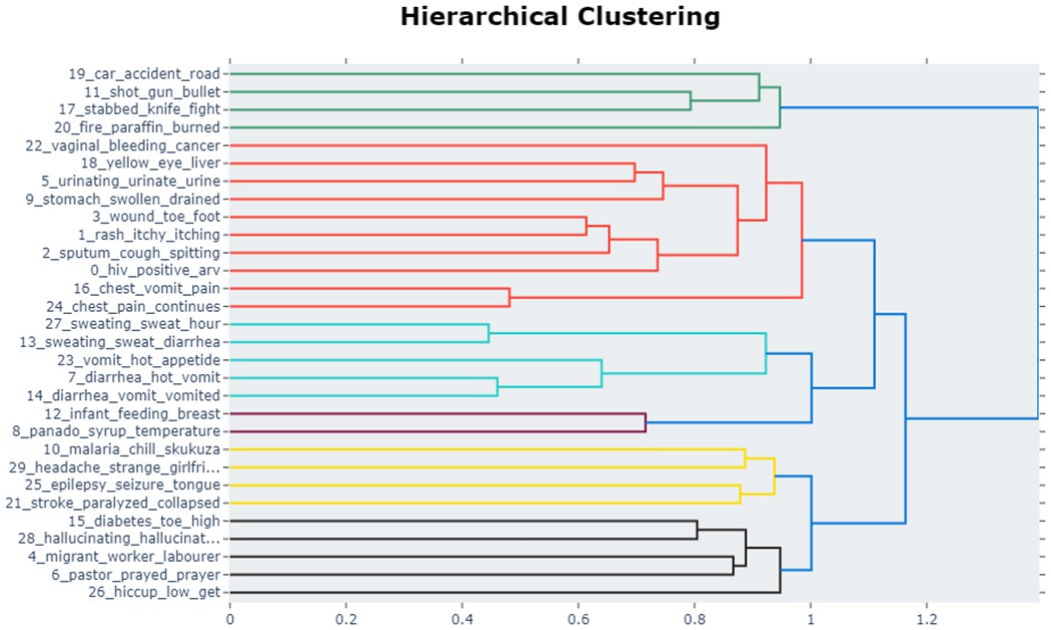
Hierarchical clusters of similar topics.

The results illustrated in Fig 8 are also visually depicted in our hierarchical clustering in Fig 10. For example, as observed from the data, the closely clustered linked topics in this group are *11, 17, 19, 20*. These are related to accident and injury mortality causes. This suggests that there were more deaths from road accidents, gender based violence and fire accidents. There is need to raise awareness to avoid the surge of such cases. Interestingly, we also note that HIV, diabetes, hypertension, TB, chronic lung and liver ailments and cervical cancer are closely clustered. This might be attributed to the fact that TB leads to lung and cancer complications. Furthermore, most HIV patients are prone to secondary diseases, that eventually worsen their condition and give rise to more deaths. Noticeably, we observe that patients with liver cirrhosis often present with urinary complaints as shown in the closely clustered topics 5 and 18. Noteworthy, is the diarrhoea cluster exhibiting typical symptoms such as *sweating, low energy, weight loss, hot body, sunken eyes*. As expected in topic 12, we note an isolated cluster on maternal related death at birth. As observed, we see that epilepsy seizures are closely linked to stroke and paralysis. As such, there is need to invest in more care workers to look after epileptic patients. Interestingly, we note that there is a high correlation on diabetic cases that result in hallucinations. Therefore, there is great need to invest in health awareness programmes, improved primary health care and early screening processes to try and curb the rise of these diseases.

### Results from the knowledge discovery process: Identified relevant topics

Our analysis suggests that most of the population in the HDSS could have died from diseases such as diarrhoea, TB, HIV, cancer, maternal, accident and injury and chronic ailments. Our LDA topic clustering shows that accident (T15) and maternal related deaths (T19) were distinct and distant from other clusters. A similar pattern and trend is also noticeable on T4 which is cervical cancer. We notice accident related deaths from the HDSS, but mostly intriguing are suicidal deaths. Of interest is cluster T1, T10 and T18. It shows that cases that succumbed to diarrhoea, respiratory tract infections, malaria and heart ailments related deaths, exhibited mostly similar symptoms. Another paramount discovery is cluster T2, T3 and T13. This cluster depicts that subjects who died from HIV also had other conditions related to TB and diabetes. Furthermore, we can also note that HIV related deaths were also associated with cancers and chronic ailments (T8, T9). T17 shows epilepsy which is also a neurological disorder. This disease is clustered mostly with T8, which is breast cancer. Additionally, we can also see that it shares some similar characteristics with HIV and TB. We can also deduce that cancer of the breast or breast infections have some common symptoms with chronic ailments of the liver, lung and kidneys as shown on the T8 and T9 cluster.

Cluster T5, T7 and T12 show that some deaths from liver ailments can lead to complications from not easy to identify symptoms which are in the unknown topics. One important finding is that HIV related cases share common symptoms with TB related deaths. From the HIV topic, through token analysis, we notice that some of the subjects were defaulting from taking their medication *stopped, refused*. Interestingly, we notice that some cases where affected by hypertension and diabetes, and this led to stroke and paralysis. This finding implies that diabetes and high blood pressure could be indicative of a form of heart metabolic syndrome. Moreover, hallucinations and mental confusion can happen with very high blood sugar levels (T7). We also discovered some cases of jaundice related deaths possibly prone in the new born babies. This calls for better controlled and effective immunisation measures in order to curb such diseases. Similar findings are also exhibited on our BERTopic analysis, as we observed from the discussions in previous paragraphs.

## Discussion

The present study’s novelty was to apply text mining techniques specifically n-gram natural language processing, LDA and BERT in topic modelling to investigate mortality causes and most prevalent diseases, using VA narrative data. As such, we managed to explore the relevancy of text mining techniques in order to discover mortality causes and the most prevalent diseases. The rich textual narratives availed valuable insights by extracting implicit knowledge on symptoms and cause of death. This is consistent with previous findings by [3]. Moreover, this study, findings are correlated with results from our previous work reported in [18, 58].

We first present the most relevant terms that were the main causes of mortality and identify the main topical diseases that were prevalent in the HDSS population. Additionally, we also managed to perform topic modelling, thus identifying interesting patterns and trends based on disease symptom similarities and differences. The generated associated disease clusters provide a statistical potential on discovering semantic level associations. Of interest, are findings from semantic and structure analysis of random cases, that show that the narratives exhibit similar and in some instances have additional symptoms as compared to those in the responses to the structured questionnaire. These findings can therefore be used to guide the health community and close the civil registration system gap. Our work also provides a methodological framework for text mining and topic clustering. Therefore, robust automated unsupervised systems that analyse VA narratives and avail interesting insights for public health improvement are vital for the community and world at large.

The findings on the n-gram natural language processing suggest that the HDSS population could have died, mostly from mortality causes such as *diarrhoea, fever, headaches, vomiting, pain, blood pressure, low energy, hot/swollen body*, amongst many. Interestingly, using topic modelling to identify most prevalent diseases, we discovered that communicable diseases such as HIV and tuberculosis were more prevalent in the HDSS. Interestingly, non communicable diseases such as diabetes, chronic ailments (kidney, heart, lung, liver), diarrhoea, cancer, respiratory tract infections, neurological disorders, hypertension and mental health ailments, were also very common in the HDSS. Expectedly, we notice distinct topics for maternal and accident and injury related deaths. This suggests that these topics have least relationships with other topics as they have dissimilar characteristics/ symptoms. A very striking observation is that neurological disorders is embedded in jaundice/ malaria/ wound with skin infection. This can be attributed to the fact that some cases had a long untreated jaundice which might have led to brain damage, a condition known as kernicterus. A similar pattern and trend is exhibited in malaria and diarrhoea and tract respiratory infections. Our topical distance map shows that HIV is central to most clusters (TB, cancer, epilepsy, diabetes, hypertension, chronic ailments), thus implying that most of the diseases exhibit symptoms as in HIV. Consequently, mortality from HIV can also be a result of complications from the earlier diseases. Noteworthy, is the fact that most people who suffer from uncontrolled hypertension and diabetes are prone to stroke and paralysis. The above findings are consistent with our initial work reported in [18, 59].

On the contrary, we note two unknown diseases, that have less informative symptoms to get to a cause of death diagnosis. However, we can see that these topics are also in the same cluster with chronic related liver ailments. Therefore, in such cases the doctors can merge the fewer symptoms in the unknowns and the ones in the known cause of death topic to probably get to a diagnosis as the symptoms are related and associated.

Our structure and semantic analyses can further provide insights that can help the public health community to extract additional implicit knowledge and symptom associations about cause of death from the large VA data. This can prove beneficial especially in cases where the physicians disagree on the cause of death and also where there is limited diagnosis information (symptoms)on the responses from the structured questionnaire. Interestingly, interpretability of CoD is made easier using our word sequence modelling approach that identifies semantics and relations in text. As such, this will save time, is cost effective and improves the diagnosis turnaround time. Of paramount importance is that, this present study methodological approach improves knowledge representation of VA textual data which supports multi disciplinary research across disciplines (See Fig 5).

Our initial study also reports on interesting findings on the most sought after treatment method when the HDSS population is faced with terminal illnesses [18]. Even though this was not the aim of this study, we notice a similar trend and pattern in the current study, as people mostly seek the traditional ways. Noteworthy, we observe diseases such as HIV, that are affecting immigrants in the HDSS. This suggests that that immigrants are also spreading such diseases. Consequently, there is great need for further investigations as this might lead to identifying subjects who default from medication and western ways of getting medical help. As such, this can possibly suggest some of the premature deaths. Informed by the study of Kabudula et al. [60], socio-economic status also plays a big role in morbidity and mortality, hence there is a great need to try address this challenge in order to improve public health.

Literature reports on the threat of non-communicable diseases and chronic diseases. Furthermore, they suggest effective implementation of primary health care systems as well as scaling up chronic based delivery, in order to curb the high mortality numbers [61]. This present study, also supports the notion raised by these authors in an effort to curb high mortality numbers.

It is imperative to improve population health, through effective intervention and support programmes, and early screening processes that are well monitored and controlled through initiatives by relevant authorities. This can prove beneficial especially for diseases such as cervical cancer.

This present study will lead to improved public health planning through the use of the findings ton inform public health strategies by identifying major health issues contributing to mortality, allowing for targeted interventions and resource allocation. Moreover, the results can be used to develop evidence-based policies addressing the specific health challenges prevalent in a given population, leading to more effective healthcare delivery. The application of topic modelling to VA data represents an innovative approach in health research, showcasing the potential of NLP in extracting valuable insights from unstructured medical narratives.

## Limitations and future work

This study was limited in complexity of the VA narratives used. The analysis entailed some terms in topics which were of little significance. This is consistent with the findings of Hacking et al. [34]. Moreover, topic segmentation has a challenge of human interpretation of discovered topics. The narratives were highly unstructured, and entailed high levels of noise, sparsity, had varying technical vocabulary terms, contain misspelled terms, had many grammatical errors, and exhibited issues around word polysemy. Nevertheless, the findings of this study still stand despite all these limitations.

Informed by the findings of this study, specifically the semantic and structure analysis, our future direction will seek to identify under-reported morbidity occurrences at the Agincourt HDSS, by effectively linking HDSS population data and NHLS public health data registries. This can further give us insights on other factors that might be leading to higher mortality numbers.

## Conclusion

The application of the unsupervised TM approaches in the current study creates a synergistic framework that leverages the strengths of each method, providing a comprehensive and accurate analysis of mortality causes and prevalent diseases within the VA narratives. The results obtained from this study contribute to the advancement of public health research by offering a sophisticated and interpretable methodology for extracting valuable insights from verbal autopsy data. As such, they can be included in the pipeline for identifying mortality causes and most prevalent diseases alongside human annotation, and interpretation. The increase in textual digital repositories can be fully utilised using automated TM approaches that can extract implicit knowledge on cause of death and avail valuable insights, which could not have been possible through manual processes.
